# Genomic insights into *Aeromonas* infections in diarrheal patients: high diversity, emerging resistance, and potential outbreak in Beijing

**DOI:** 10.3389/fmicb.2026.1909810

**Published:** 2026-08-24

**Authors:** Aixia Yan, Yuwei Liu, Shoufei Li, Xinmiao Wu, Jie Yang, Yameng Gao, Yu Zhao, Luotong Wang, Bo Pang, Ying Li

**Affiliations:** 1 Shunyi District Center for Disease Control and Prevention, Beijing, China; 2 National Institute for Communicable Disease Control and Prevention, Chinese Center for Disease Control and Prevention, Beijing, China

**Keywords:** *Aeromonas*, antimicrobial resistance, diarrhea, foodborne pathogen, *mcr* gene, outbreak, virulence factors, whole-genome sequencing

## Abstract

**Background:**

*Aeromonas* species are ubiquitous aquatic bacteria that have emerged as significant foodborne enteric pathogens worldwide, yet their genomic landscape in clinical settings remains poorly delineated, particularly in Beijing.

**Methods:**

To address this gap, we performed active surveillance for *Aeromonas* among 691 consecutive diarrheal outpatients in a Beijing district from January to December 2024. Isolates were recovered using enrichment culture coupled with PCR screening and identified by MALDI-TOF MS. Antimicrobial susceptibility was tested against 17 agents. Whole-genome sequencing was conducted on all isolates, enabling average nucleotide identity (ANI) analysis, comprehensive annotation of antimicrobial resistance and virulence genes, multilocus sequence typing (MLST), and core-genome SNP (cgSNP)-based phylogenetics.

**Results:**

*Aeromonas* was detected in 3.3% (23/691) of patients, with *Aeromonas veronii* (56.52%, 13/23) and *Aeromonas caviae* (26.09%, 6/23) predominating. Co-infections with other enteric pathogens occurred in 65.2% of positive cases. Resistance rates were notably high for ampicillin/ampicillin-sulbactam (78.26%), nalidixic acid (56.52%), and ertapenem (21.73%), and 65.21% of isolates were multidrug-resistant. Genotypic–phenotypic concordance was robust, with *β*-lactamase genes *ampS* (60.87%) and *blaCEPH-A3* (47.83%) being most prevalent. Strikingly, *mcr-3.25* and *mcr-3.3*, which belong to the *mcr* family (originally described as mobile colistin resistance genes), were identified in 8.70% of isolates, exhibiting perfect correlation with phenotypic resistance. Plasmer analysis suggested both *mcr* genes to be chromosomally encoded. Comparative genomic analysis of virulence-associated genes revealed striking species-specific specialization: *A. veronii* predominantly carried complete T3SS clusters (61.5%), *A. caviae* and *Aeromonas enteropelogenes* were enriched in T6SS genes, and a single *A. dhakensis* isolate possessed an extensive arsenal including T3SS, T6SS, and a full RTX toxin cluster. MLST resolved the 23 isolates into 22 sequence types, 18 of which were novel. Phylogenetic reconstruction identified a tight monophyletic cluster of three *A. veronii* isolates (9–27 SNP differences) recovered within a 96-h window, suggestive of a potential cluster that warrants further epidemiological investigation. Comparative genomic analysis of the rare species *Aeromonas allosaccharophila* demonstrated that the Beijing clinical isolate S14 differs from the U.S. clinical strain ATCC 35942 by 42,099 SNPs, confirming its distinct genetic lineage.

**Conclusion:**

Collectively, this study delineates high genetic diversity, emerging chromosomal colistin resistance, and species-specific virulence specialization among *Aeromonas* isolates from diarrheal patients in Beijing. The detection of a potential outbreak cluster and a rare clinical isolate underscores the power of genomics-based surveillance for detecting and mitigating foodborne pathogen thre*ats*.

## 1 Introduction

*Aeromonas*, Gram-negative bacteria ubiquitous in aquatic environments, have increasingly been recognized as an emerging human pathogen. Despite this, our understanding of *Aeromonas* diversity, especially the relationship between clinical and environmental strains, remains limited (Singh et al., 2026). Globally, reported detection rates of *Aeromonas* in diarrheal patients vary considerably (Sadeghi et al., 2024; Lou et al., 2025). A systematic review and meta-analysis of global spread and antimicrobial resistance of *Aeromonas hydrophila* in aquatic food animals highlighted the widespread distribution of this pathogen (Jeamsripong et al., 2025). In China, a study from Wenzhou reported a detection rate of 2.24% (Lou et al., 2025). The genus *Aeromonas* is widely distributed in aquatic environments and has been described in association with human infections, with most cases caused by *Aeromonas caviae*, *Aeromonas veronii*, and *A. hydrophila*. More recently, *Aeromonas dhakensis* has emerged as an increasingly important human pathogen (Calvo Sánchez et al., 2025).

Pathogenesis involves a diverse arsenal of virulence factors. Epidemiological studies have reported that genes encoding the type III secretion system (T3SS) are highly prevalent in *A. veronii* and *A. hydrophila* clinical isolates (Chacón et al., 2004; Silver and Graf, 2009). Conversely, a recent global genomic analysis revealed that strains carrying the type VI secretion system (T6SS) are significantly more common in *A. caviae* isolated from human gastroenteritis cases than from environmental sources (Chong et al., 2024). Notably, *A. dhakensis* carries an extensive virulence repertoire and has been associated with more severe clinical manifestations (Chen et al., 2016). This finding aligns with a recent in silico genomic analysis conducted on Brazilian *Aeromonas jandaei* strains, which identified significant virulence mechanisms, including T2SS, T3SS, and notably T6SS (Duarte et al., 2025).

Compounding these virulence concerns, antimicrobial resistance in *Aeromonas* has become a growing challenge. Recent studies report rising resistance to *β*-lactams, carbapenems, and fluoroquinolones (Chen et al., 2014; Sun et al., 2021). Of particular concern is the emergence of plasmid-mediated colistin resistance genes (*mcr*) in Gram-negative bacteria (Liu et al., 2016; Shen et al., 2018). Critically, emerging evidence has revealed that *Aeromonas* species serve as natural reservoirs of *mcr-3* family genes, harboring nonmobile colistin resistance (*NMCR-3*) determinants on their chromosomes that represent the ancestral precursors of plasmid-borne *mcr-3* (Yu et al., 2023). This evolutionary relationship positions *Aeromonas* as a critical sentinel for monitoring colistin resistance mobilization.

In Beijing, our research group has conducted continuous *Aeromonas* surveillance since 2016. A previous study based on archived isolates from adult diarrhea patients and aquatic environments during 2016–2022 reported a 0.65% isolation rate and identified *A. veronii* and *A. caviae* as predominant species (Li et al., 2025). Patient-derived isolates exhibited high resistance to ampicillin and ampicillin-sulbactam, with 61.5% classified as multidrug-resistant. Phylogenetic analysis revealed intertwined distribution of clinical and environmental isolates, suggesting potential transmission links between aquatic reservoirs and human infections (Li et al., 2025). However, that study was limited by reliance on passively collected isolates and conventional methods that lacked resolution for precise species discrimination and distinction between chromosomal versus plasmid-borne resistance determinants—a gap with critical implications for understanding transmission risk.

Building upon this foundational work, we conducted active surveillance for *Aeromonas* among diarrheal patients in a Beijing district throughout 2024, employing systematic enrollment, enhanced detection methods (enrichment culture with PCR screening), and whole-genome sequencing. This approach enabled precise species classification via ANI analysis, comprehensive resistance and virulence gene profiling, and determination of the chromosomal localization of the detected *mcr* genes. This study aims to elucidate species distribution, antimicrobial resistance profiles (with particular attention to *mcr* genes and their genomic context), virulence gene repertoires, and population structure of circulating *Aeromonas* strains, including a detailed global genomic comparison of the rare clinical isolate *Aeromonas allosaccharophila*, to inform evidence-based control strategies for this emerging foodborne pathogen in China.

## 2 Materials and methods

### 2.1 Study design and sample collection

A laboratory-based active surveillance study was conducted from January to December 2024 in a district of Beijing, China. A total of 691 fecal samples were collected from consecutive outpatients presenting with acute diarrhea, defined as the passage of three or more loose, watery, mucoid, or bloody stools within a 24 h period, at three sentinel hospitals. Samples were placed in Cary-Blair transport medium, stored at 4 °C, and transported to the reference laboratory within 24 h. Demographic information, including age and sex, was recorded for epidemiological analysis.

### 2.2 *Aeromonas* isolation and identification

Fecal samples (200 mg) were inoculated into alkaline peptone water (APW) and incubated at 37 °C for 24 h. The enrichment culture was then streaked onto MacConkey agar and Rimler-Shotts selective agar, followed by incubation at 37 °C for 24 h. Presumptive *Aeromonas* colonies (lactose-negative on MacConkey agar) were subcultured on tryptic soy agar to obtain pure isolates. Genus-level confirmation was performed by fluorescent PCR targeting the *Aeromonas* 16S rRNA gene (Du et al., 2020). Species-level identification was carried out using MALDI-TOF MS (Bruker).

### 2.3 Antimicrobial susceptibility testing

Antimicrobial susceptibility testing was performed using the broth microdilution method according to the Clinical and Laboratory Standards Institute (CLSI) guideline M45A2E (CLSI, 2019). *Escherichia coli* ATCC 25922 was used as the quality control strain. The following 17 antimicrobial agents were tested: ampicillin (AMP), ampicillin-sulbactam (AMS), tetracycline (TET), meropenem (MEM), polymyxin E (CT), ertapenem (ETP), ceftazidime/avibactam (CZA), tigecycline (TGC), cefotaxime (CTX), ceftazidime (CAZ), ciprofloxacin (CIP), azithromycin (AZI), chloramphenicol (CHL), nalidixic acid (NAL), streptomycin (STR), trimethoprim-sulfamethoxazole (SXT), and amikacin (AMK). Multidrug resistance was defined as acquired non-susceptibility to at least one agent in three or more antimicrobial categories.

### 2.4 Whole-genome sequencing

Genomic DNA was extracted from overnight bacterial cultures using the Wizard Genomic DNA Purification Kit (Promega, Madison, WI, United States). DNA libraries were prepared with the Illumina DNA Prep Kit and sequenced on an Illumina HiSeq platform (2 × 150 bp paired-end reads). Raw sequencing reads were quality-controlled using FastQC v0.11.9, and adapter sequences were trimmed with Trimmomatic v0.39. Denovo genome assembly was performed with SPAdes v3.15.5 using default parameters. Assembly quality was assessed with QUAST v5.2.0.

### 2.5 Bioinformatic analyses for species identification, MLST, and phylogeny

Species identification was performed using FastANI v1.33 with a 95% average nucleotide identity (ANI) threshold against reference type strain genomes (*A. hydrophila* ATCC 7966 T [accession No.: GCA_000014805], *A. caviae* NCTC 12244 T [accession No.: GCA_900476005], *A. dhakensis* TN14 [accession No.: GCA_905132925], *A. veronii* FDAARGOS_632 [accession No.: GCA_008693705], *A. media* CECT 4232 T [accession No.: GCA_000819985], *Aeromonas sanarellii* LMG 24682 T [accession No.: GCA_000820085], *A. schubertii* ATCC 43700 T [accession No.: GCA_001481395], *A. allosaccharophila* FDAARGOS_933 [accession No.: GCA_016026615], *A. enteropelogenes* FDAARGOS_1510 [accession No.: GCA_020097295]). Multilocus sequence typing (MLST) was performed using the *Aeromonas* PubMLST scheme.^1^ For phylogenetic analysis of the 23 clinical isolates, core-genome single nucleotide polymorphisms (cgSNPs) were identified using Snippy v4.6.0. Recombination regions were removed with Gubbins v2.4.1, and a maximum-likelihood phylogenetic tree was constructed using IQ-TREE v2.2.0 with 1,000 ultrafast bootstrap replicates.

### 2.6 Antimicrobial resistance and virulence gene annotation

Antimicrobial resistance genes and virulence factors were identified using ABRicate v1.0.1 against the Comprehensive Antibiotic Resistance Database (CARD) and the Virulence Factor Database (VFDB), respectively, with minimum thresholds of 95% identity and 95% coverage. For isolates carrying *mcr* genes, chromosomal versus plasmid localization was determined using Plasmer (version 1.0, https://github.com/zhenchengfang/Plasmer), a bioinformatic tool that distinguishes plasmid-derived from chromosome-derived contigs in bacterial genome assemblies, with default parameters. To evaluate the genetic conservation of the *mcr* flanking regions, the complete contigs harboring the respective *mcr* genes were extracted from the genome assemblies. Pairwise sequence alignment was performed using BLASTn (v2.12.0) to determine nucleotide identity, query coverage, and the presence of insertions or deletions between the two contigs. Insertion sequence (IS) elements within the flanking regions were identified using the ISfinder database^2^ through BLASTn searches against the ISfinder reference collection.

### 2.7 Comparative genomic analysis of *Aeromonas allosaccharophila*

To place the first Chinese clinical isolate of *A. allosaccharophila* (S14) in a global context, a systematic search of the NCBI Assembly database (accessed March 2025) was conducted using the taxonomy ID for *A. allosaccharophila* (taxid: 656). All complete or draft genome assemblies were retrieved. Six representative genomes, each with a unique NCBI accession number, were selected for detailed analysis: the U.S. clinical strain ATCC 35942 (GCA_006246445.1), the environmental isolate BVH88 (GCA_000820245.1), the type strain CECT 4199 (GCA_000819685.1), the complete genome FDAARGOS_933 (GCA_016026615.1), the strain Z9-6 (GCA_900491725.1), and the fish isolate BIM B-1809 (GCA_032849865.1). A full list of all publicly available *A. allosaccharophila* genomes is provided in Supplementary Table S1. For phylogenetic reconstruction, cgSNPs were identified using Snippy v4.6.0 with the complete genome of FDAARGOS_933 as the reference. Recombination regions were removed using Gubbins v2.4.1, and a maximum-likelihood tree was constructed with IQ-TREE v2.2.0 under the GTR + F + I + G4 substitution model (selected by ModelFinder) and 1,000 ultrafast bootstrap replicates. The tree was visualized with iTOL v6. A core-genome SNP distance matrix was calculated using snp-dists v0.8.2. For the main text, a subset of six representative strains was used to highlight key phylogenetic relationships; the full tree incorporating all available genomes (*n* = 57) is provided as Supplementary Figure S1.

### 2.8 Statistical analysis

Statistical analyses were performed using SPSS software version 25.0. The chi-square test was used to compare isolation rates among different groups (sex, age, season). A *p*-value < 0.05 was considered statistically significant.

## 3 Results

### 3.1 Epidemiological and clinical characteristics of *Aeromonas* infection

Among 691 diarrheal patients, 23 confirmed *Aeromonas* isolates were obtained, yielding a detection rate of 3.3% (23/691). No significant difference in detection rates was observed between males (2.5%, 10/393) and females (4.4%, 13/298) (χ^2^ = 1.741, *p* > 0.05) (Table 1). Seasonal distribution showed peak isolation rates in May (5.2%, 5/96), August (4.6%, 5/108), and April (4.5%, 3/67), with no isolates recovered from November through March. Patient ages ranged from 19 days to 75 years, with detection rates comparable *acr*oss age groups (*p* > 0.05). Co-infections with other enteric pathogens—including *diarrheagenic Escherichia coli*, *Salmonella*, *Campylobacter*, *norovirus*, and *rotavirus*—were identified in 65.2% (15/23) of *Aeromonas*-positive individuals. All patients presented with diarrhea as the primary symptom; stool characteristics were watery (60.9%, 14/23), loose (26.1%, 6/23), and bloody (4.3%, 1/23). Accompanying clinical manifestations included fever (29.2%, 7/23), nausea (25.0%, 6/23), and vomiting (20.8%, 5/23) (Table 1).

**Table 1:** Epidemiological and clinical characteristics of 23 *Aeromonas*-positive patients with diarrhea (*n* = 23).

Patient number	Sex	Age	Clinical symptoms	Pathogens detected	Strain number
P1	Male	36 years	diarrhea (Watery stools 10 times per day) + abdominal pain + fatigue + fever (38.5 °C)	*A. veronii* + EPEC + *Clostridium perfringens* + *Rotavirus*	S1
P2	Female	36 years	diarrhea (Watery stools 8 times per day) + abdominal pain + nausea	*A. veronii* + EAEC + *Clostridium perfringens* + *Rotavirus*	S2
P3	Female	41 years	diarrhea (Watery stools 10 times per day) + nausea + fever (39.0 °C)	*A. veronii* + *Campylobacter*	S3
P4	Male	26 years	diarrhea (Watery stools 10 times per day) + abdominal pain + nausea + vomiting	*A. veronii* + EPEC + *Sapovirus*	S4
P5	Female	75 years	diarrhea (Watery stools 10 times per day) + abdominal pain	*A. caviae*	S5
P6	Male	37 years	diarrhea (Watery stools 10 times per day) + abdominal pain + fever (38.5 °C)	*A. veronii* + *Norovirus* + *Astrovirus*	S6
P7	Female	35 years	diarrhea (Watery stools 6 times per day) + abdominal pain	*A. veronii*	S7
P8	Female	51 years	diarrhea (Watery stools 10 times per day) + abdominal pain + fatigue + nausea + vomiting	*A. caviae* + *Shewanella* + *Norovirus*	S8
P9	Female	73 years	diarrhea (Watery stools 10 times per day) + abdominal pain + fever (38.3°C)	*A. caviae* + *Salmonella*	S9
P10	Male	29 years	diarrhea (Watery stools 6 times per day)	*A. veronii* + *Campylobacter*	S10
P11	Male	44 years	diarrhea (Watery stools 6 times per day) + fever (39.0 °C)	*A. enteropelogenes* + *Shewanella* + *Norovirus*	S11
P12	Male	20 years	diarrhea (Watery stools 10 times per day) + abdominal pain + fever (37.6°C)	*A. veronii*	S12
P13	Male	70 years	diarrhea (Watery stools 6 times per day) + abdominal pain + nausea	*A. caviae*	S13
P14	Female	7 years	diarrhea (Loose stools 3 times per day)	*A. allosaccharophila* + *Norovirus*	S14
P15	Female	2 years	vomiting	*A. enteropelogenes*	S15
P16	Male	3 months	diarrhea (Loose stools 3 times per day)	*A. veronii*	S16
P17	Male	8 months	diarrhea (Loose stools 8 times per day)	*A. caviae*	S17
P18	Female	7 years	diarrhea (Loose stools 8 times per day)	*A. veronii*	S18
P19	Female	36 years	diarrhea (bloody stool 8 times per day)	*A. veronii* + *Salmonella*	S19
P20	Male	14 years	diarrhea (loose stool 10 times per day) + fever (37.9 °C)	*A. veronii* + *Salmonella* + *Campylobacter*	S20
P21	Female	2 years	diarrhea (loose stool 3 times per day)	*A. veronii* + *Salmonella* + *Clostridium perfringens*	S21
P22	Female	8 years	diarrhea (Watery stools 10 times per day) + fever (37.9 °C)	*A. caviae* + *Salmonella*	S22
P23	Female	1 months	diarrhea (Watery stools 6 times per day)	*A. dhakensis* + *Rotavirus*	S23

### 3.2 Genomic characteristics, species composition, and MLST analysis

**Figure 1:**
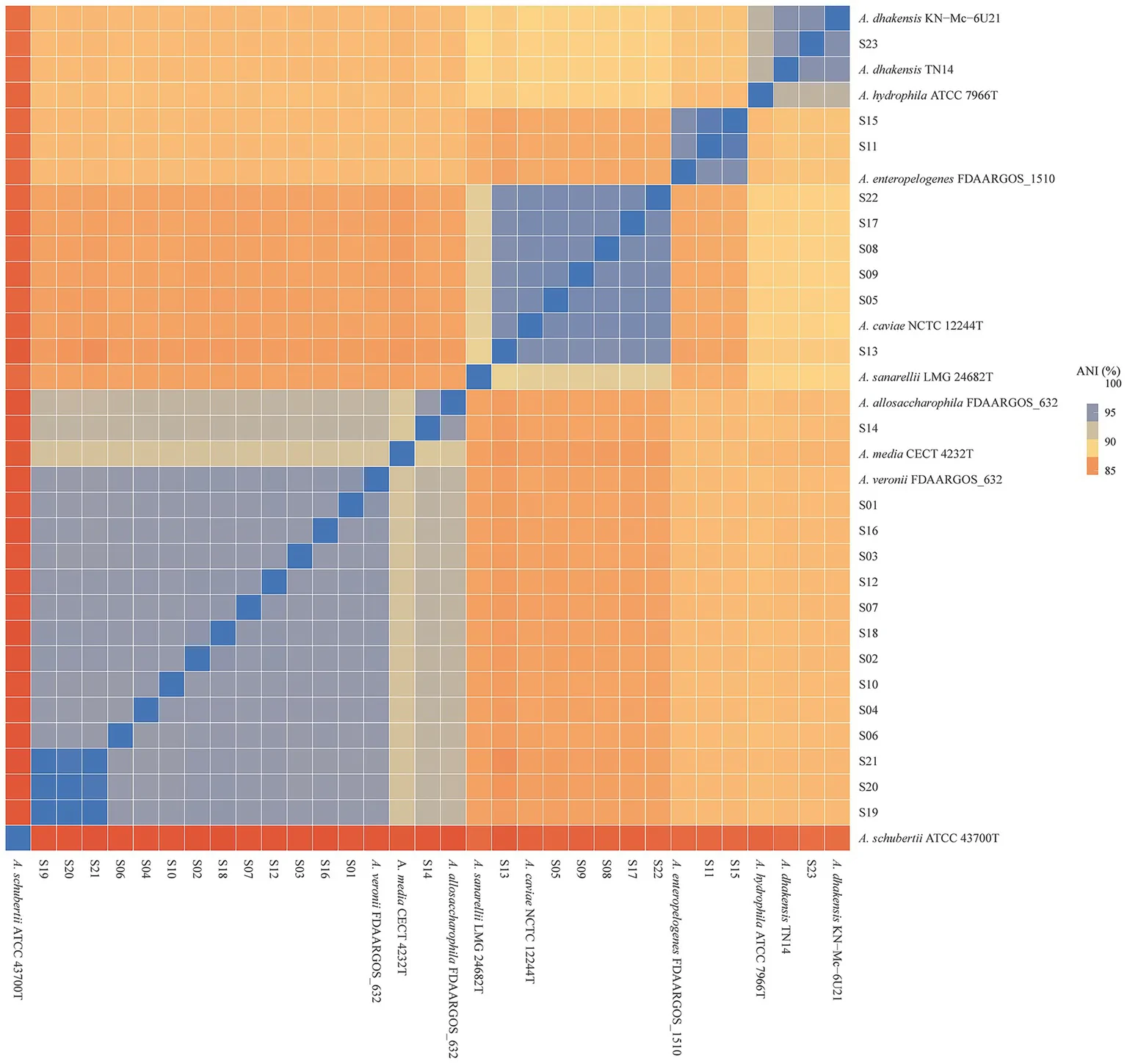
Average nucleotide identity (ANI) analysis of 23 *Aeromonas* isolates from diarrheal patients in Beijing, 2024. Heatmap showing pairwise ANI values against reference type strains. Nine type strains (*A. hydrophila* ATCC 7966, *A. caviae* NCTC 12244, *A. dhakensis* TN14, *A. veronii* FDAARGOS_632, *A. media* CECT 4232, *A. sanarellii* LMG 24682, *A. schubertii* ATCC 43700, *A. allosaccharophila* FDAARGOS_933, *A. enteropelogenes* FDAARGOS_1510) were used as reference genomes. The color from yellow to blue represents the ANI values from 80 to 100%.

**Figure 2:**
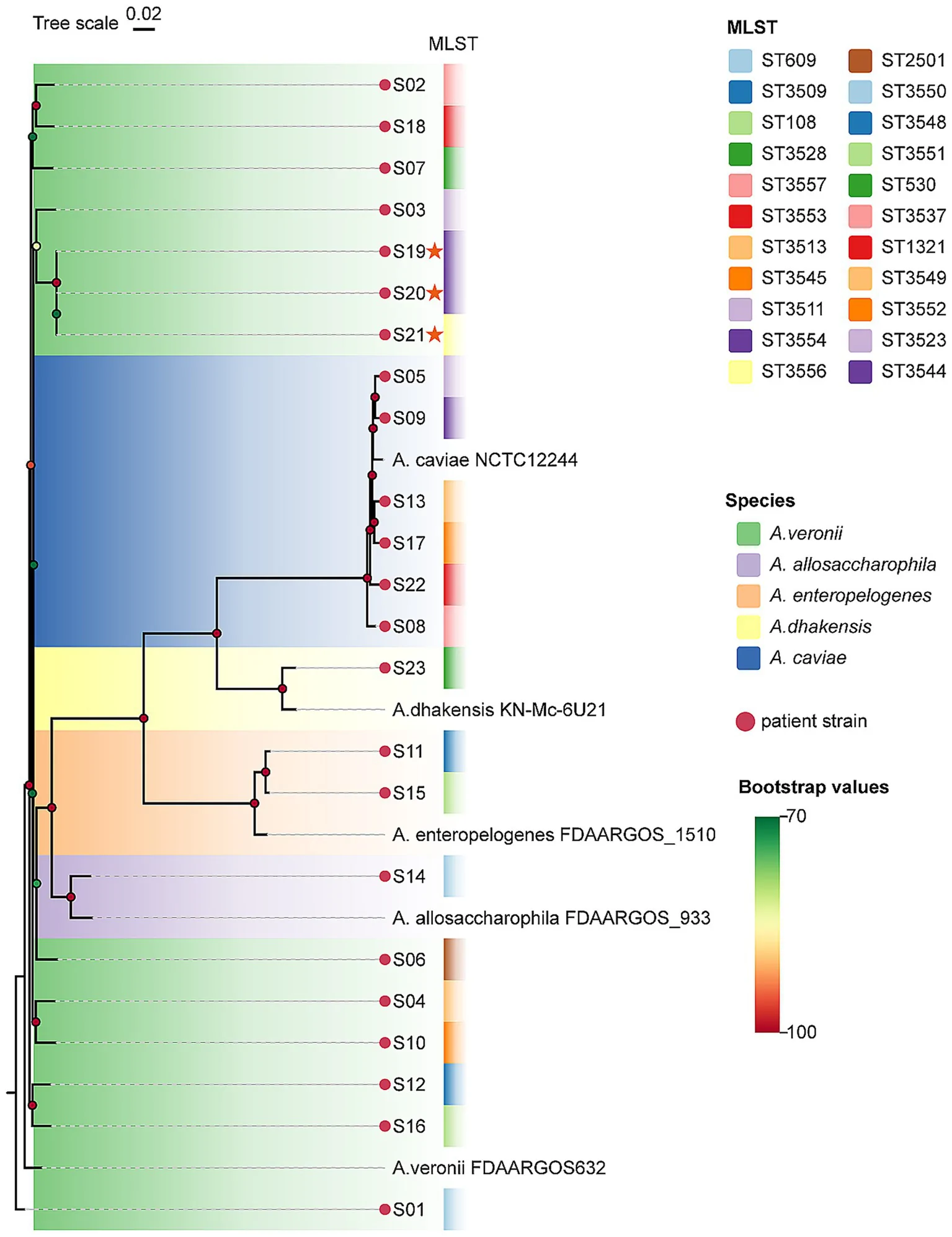
Maximum-likelihood phylogenetic tree based on core-genome single nucleotide polymorphisms (cgSNPs) of 23 *Aeromonas* isolates. Reference genomes are included for taxonomic context. The tree reveals four distinct species-specific clades. Red dots indicate patient-derived isolates. The tight cluster of three *A. veronii* isolates (S19, S20, S21) is highlighted. Bootstrap values (70–100%) are indicated by branch color gradient from red to green.

Whole-genome sequencing of all 23 isolates yielded high-quality draft genomes, with a mean size of 5.17 Mbp, an average contig count of 59, an N50 of 360.5 kbp, and a mean GC content of 45.21%. Average nucleotide identity (ANI) analysis against reference genomes revealed a diverse species composition: *A. veronii* was the most prevalent (56.52%, 13/23), followed by *A. caviae* (26.09%, 6/23), *A. enteropelogenes* (8.70%, 2/23), *A. dhakensis* (4.35%, 1/23), and *A. allosaccharophila* (4.35%, 1/23) (Figure 1). Multilocus sequence typing (MLST) revealed substantial genetic diversity, with the 23 isolates assigned to 22 distinct sequence types (STs), including 18 novel STs (Figure 2).

### 3.3 Antimicrobial resistance profiles and genetic determinants

**Figure 3:**
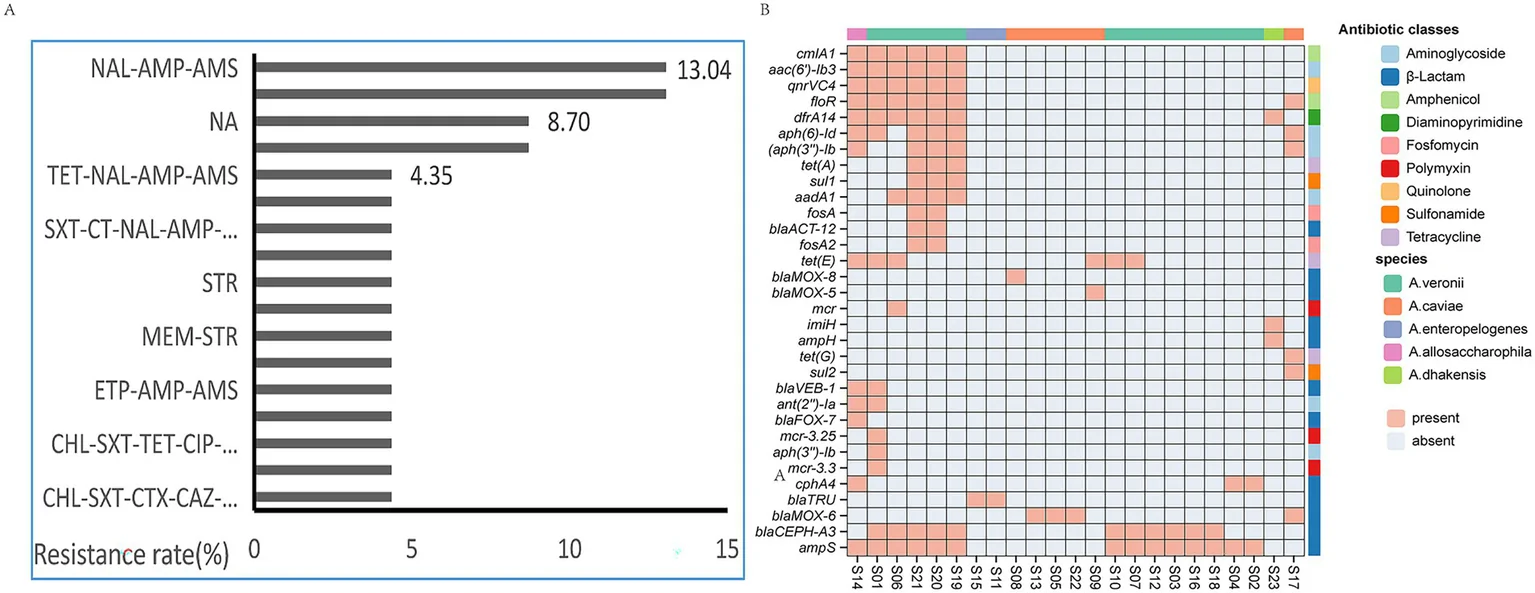
Antimicrobial resistance profiles of 23 *Aeromonas* isolates. **(A)** Heatmap showing phenotypic resistance patterns to 17 antibiotics. **(B)** Distribution of antimicrobial resistance genes across isolates by drug class. AMP, ampicillin; AMS, ampicillin-sulbactam; TET, tetracycline; MEM, meropenem; CT, polymyxin E; ETP, ertapenem; CZA, ceftazidime/avibactam; TGC, tigecycline; CTX, cefotaxime; CAZ, ceftazidime; CIP, ciprofloxacin; AZI, azithromycin; CHL, chloramphenicol; NAL, nalidixic acid; STR, streptomycin; SXT, selectrin; AMK, amikacin.

Phenotypic antimicrobial susceptibility testing revealed high resistance rates to ampicillin and ampicillin-sulbactam (78.26%, 18/23), nalidixic acid (56.52%, 13/23), streptomycin (34.78%, 8/23), tetracycline (17.39%, 4/23), ertapenem (21.73%, 5/23), and trimethoprim-sulfamethoxazole (21.73%, 5/23). Lower resistance rates were observed for chloramphenicol (13.04%, 3/23), meropenem (8.70%, 2/23), colistin (8.70%, 2/23), ciprofloxacin (8.70%, 2/23), cefotaxime (4.35%, 1/23), and ceftazidime (4.35%,1/23). All isolates remained fully susceptible to ceftazidime-avibactam, tigecycline, azithromycin, and amikacin. Multidrug resistance (MDR) was observed in 65.21% (15/23) of isolates, with the dominant resistance patterns being AMP + AMS and NAL + AMP + AMS, each accounting for 13.04% of isolates. The phenotypic resistance profiles of individual isolates are shown in Figure 3A.

**Figure 4:**
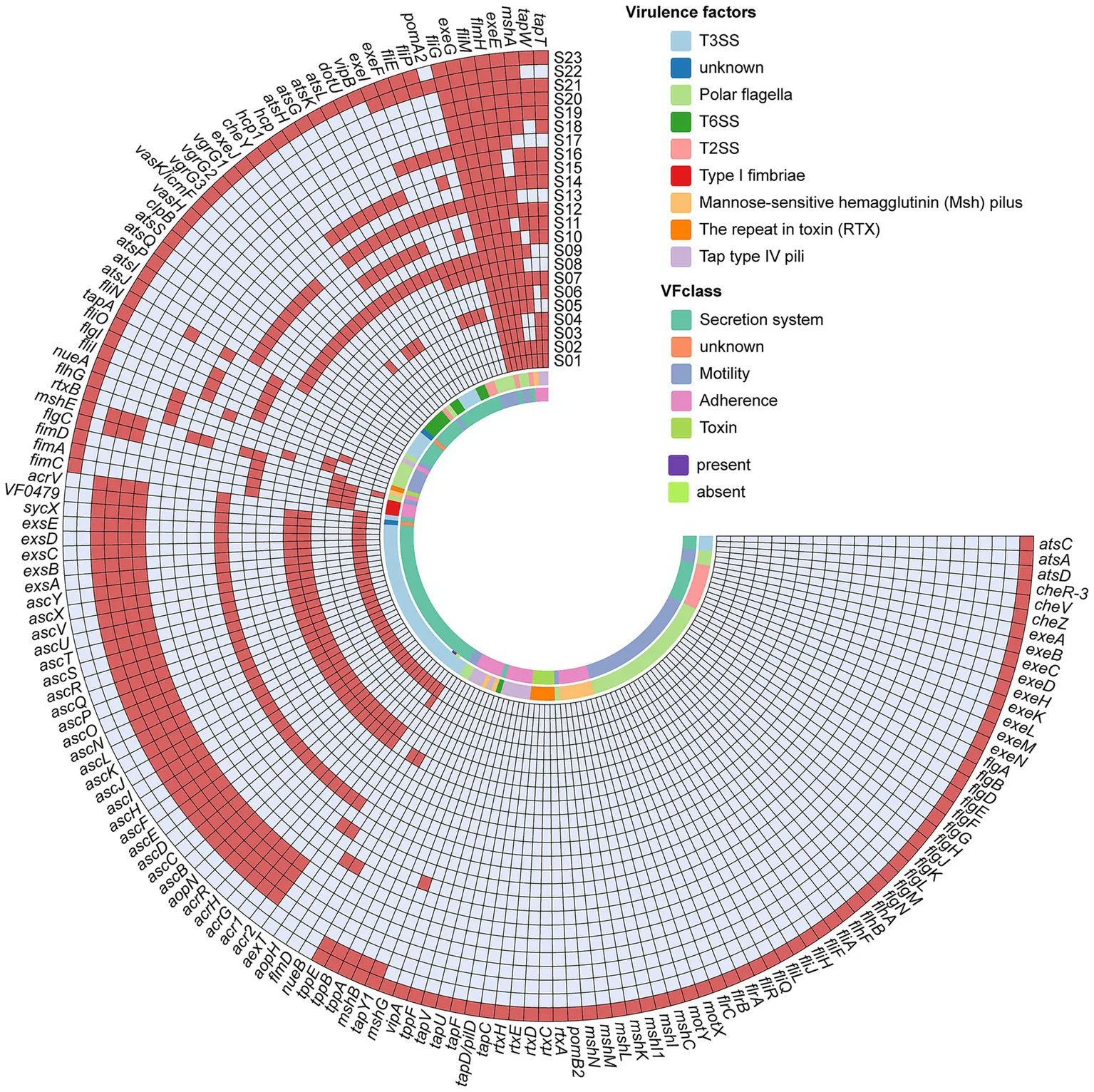
Virulence gene distribution among 23 *Aeromonas* isolates. Heatmap showing presence/absence of key virulence factors associated with secretion systems, adhesion, motility, and toxicity.

Whole-genome analysis identified 32 antimicrobial resistance genes (ARGs) across 9 drug classes (Figure 3B). The three most prevalent ARGs were the *β*-lactamase genes *ampS* (60.87%, 14/23) and *blaCEPH-A3* (47.83%, 11/23), followed by the trimethoprim resistance gene *dfrA14* (30.43%, 7/23) and the chloramphenicol resistance gene *floR* (30.43%, 7/23). Carbapenemase genes (*blaMOX-5*, *blaMOX-6*, *blaMOX-8*, and *imiH*) were detected in 30.43% (7/23) of isolates, and their presence was fully concordant with phenotypic carbapenem resistance. Five aminoglycoside-modifying enzyme genes were identified: *aadA1* (17.39%, 4/23), *aph(6)-Id* (26.09%, 6/23), *aac(6′)-Ib3* (26.09%, 6/23), *aph(3″)-Ib* (21.74%, 5/23), and *ant(2″)-Ia* (8.70%, 2/23). The plasmid-mediated quinolone resistance gene *qnrVC4* was present in 26.09% (6/23) of isolates. Tetracycline resistance genes (*tet(A), tet(E),* and *tet(G)*) were carried by 43.48% (10/23) of isolates, while the sulfonamide resistance genes *sul1* and *sul2* were detected in 13.0% (3/23) and 4.3% (1/23) of isolates, respectively. Notably, *mcr* genes were predicted in 8.70% (2/23) of isolates, showing high concordance with phenotypic colistin resistance (both strains with *mcr* genes were among the two colistin-resistant isolates). Using Plasmer analysis, both *mcr* genes were predicted to be located on the chromosome. The contig from strain S1 was 1,468 bp, covering the *mcr* gene with limited flanking extensions, whereas the contig from strain S6 was 14,370 bp, providing complete flanking sequences. BLASTn alignment revealed that the S1 contig showed 100% query coverage and 99.80% nucleotide identity (1,465/1468 identical sites) with the corresponding region in S6, with no insertions or deletions detected. ISfinder annotation identified a complete ISAeca1 transposase (IS30 family) (Supplementary Figure S2). No resistance genes associated with ceftazidime-avibactam, tigecycline, or amikacin were detected, consistent with the full susceptibility observed phenotypically (see Figure 4).

### 3.4 Virulence factor distribution

Comprehensive virulence gene annotation identified 153 factors associated with adhesion, secretion, motility, and toxicity. Among the 23 *Aeromonas* isolates, all strains universally carried the type II secretion system (T2SS) core gene *exeG* and the polar flagella core genes *fliG*, *fliM*, and *flmH* (100% positivity). The *acr/exs*-type T3SS cluster (including *acr1, acr2, acrG-H, exsA-E, sycX, VF0479*) was detected exclusively in eight of the 13 *A. veronii* isolates (61.5%; S2, S6, S7, S12, S18, S19, S20, S21). The *ats*-type T3SS cluster, defined by the signature gene *atsA*, was found only in the single *A. dhakensis* isolate (S23). Other *ats* family genes (e.g., *atsG, atsH, atsK, atsL*) were detected in two *A. caviae* (S9, S13) and two *A. enteropelogenes* (S11, S15), but the complete *ats* cluster (*atsA*-positive) was absent from these strains. The core T6SS gene *hcp* was present in six isolates: three *A. caviae* (S5, S9, S13), two *A. enteropelogenes* (S11, S15), and one *A. dhakensis* (S23). No *A. veronii* isolate carried *hcp*. The RTX toxin gene cluster (*rtxA-E*, *rtxH*) was unique to *A. dhakensis* (S23). Type I fimbriae genes (*fimA/C/D*) were detected in three *A. veronii* isolates (S3, S4, S10) and in *A. dhakensis* (S23). *Mannose-sensitive hemagglutinin* (*Msh*) *pilus* genes and Tap type IV pilus genes showed scattered distribution, with the most complete repertoires again observed in *A. dhakensis* (S23). The single *A. allosaccharophila* isolate (S14) possessed a limited virulence repertoire, lacking T3SS, T6SS, RTX, and most adherence systems. Considerable intraspecies heterogeneity was evident: among the 13 *A. veronii* isolates, eight carried *acr/exs*-type T3SS and the remaining five lacked both T3SS and T6SS, carrying only the core T2SS and *flagellar* genes. In *A. caviae* (*n* = 6), three carried T6SS and none carried T3SS. Both *A. enteropelogenes* isolates (*n* = 2) carried T6SS but not T3SS. *A. dhakensis* (*n* = 1) possessed the most comprehensive virulence repertoire, including *ats*-type T3SS, T6SS, RTX, and multiple adhesins.

### 3.5 Population genomics and molecular epidemiology

Core-genome SNP-based phylogenetic analysis segregated the 23 isolates into four distinct species-specific clades, with inter-clade genetic distances ranging from 13,296 to 49,586 SNPs. Within the *A. veronii* clade, three isolates (S19, S20, and S21), each recovered from a different patient, formed a tight monophyletic cluster, differing by only 9–27 SNPs. Two of these isolates shared the same novel sequence type ST3554 and were recovered within a 96-h window, suggesting a potential localized cluster that merits further investigation (Figure 2).

### 3.6 Global genomic comparison of the *Aeromonas allosaccharophila* clinical isolate

**Figure 5:**
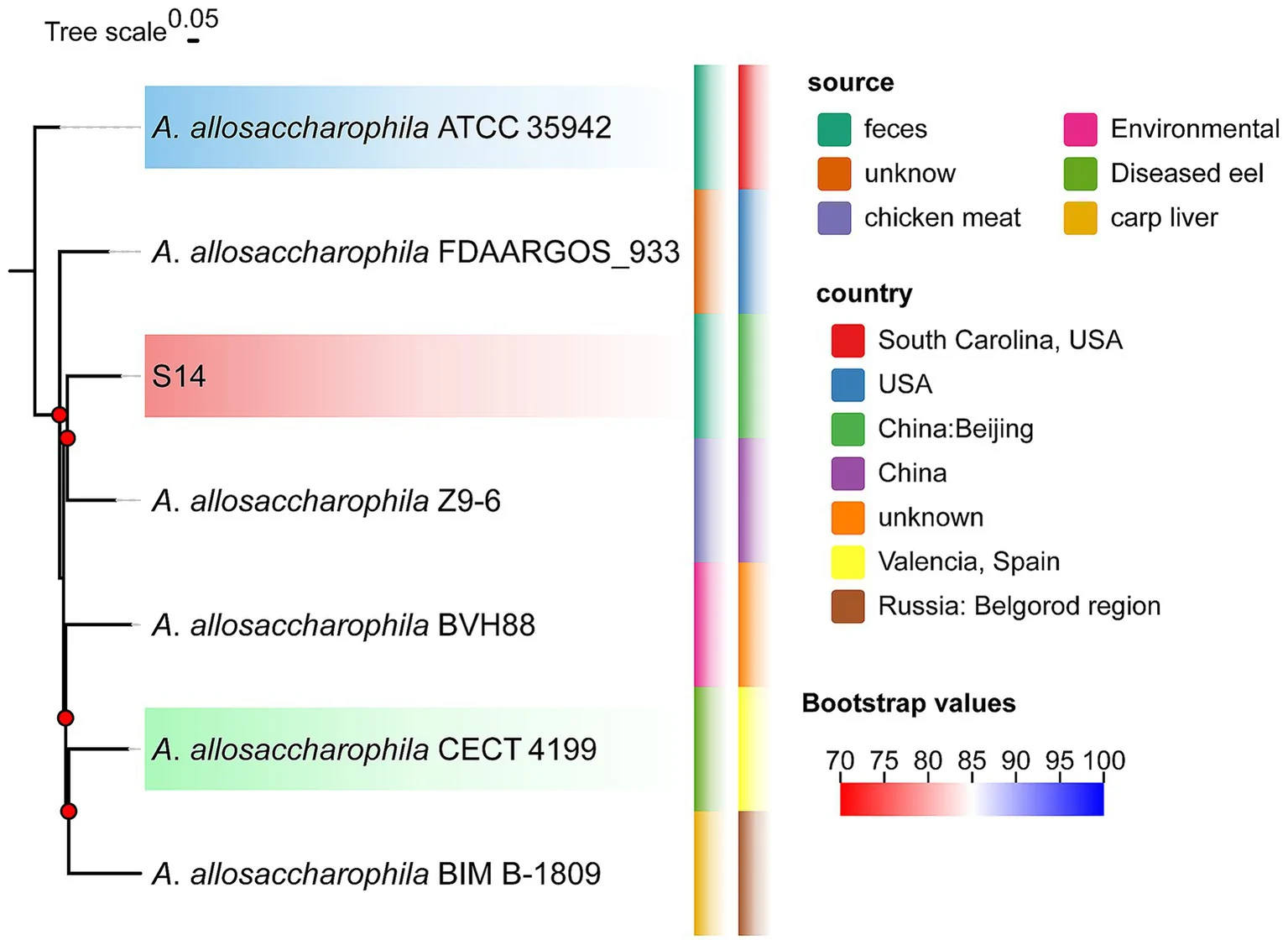
Core-genome SNP-based phylogenetic tree of the Beijing clinical isolate S14 and six representative publicly available *Aeromonas allosaccharophila* genomes. S14 (this study) is highlighted in red; the U.S. clinical isolate ATCC 35942 is highlighted in blue; the type strain CECT 4199 is shown in green. The tree demonstrates that S14 forms a distinct branch separate from all other strains, with substantial genetic distance from the U.S. clinical isolate (42,099 SNPs).

To investigate the genetic relationship of the Beijing isolate S14 within the global *A. allosaccharophila* population, we constructed a core-genome SNP-based phylogeny using six representative publicly available genomes (Figure 5). The phylogenetic tree showed that S14 formed a distinct branch separate from all other strains. The two human clinical isolates, S14 (China, 2024) and ATCC 35942 (USA, 1992), did not cluster together; instead, they were separated by multiple environmental strains. The core-genome SNP distance matrix (Supplementary Table S2) showed that S14 differed from the U.S. clinical isolate ATCC 35942 by 42,099 SNPs, from the type strain CECT 4199 by 44,223 SNPs, from the complete genome reference FDAARGOS_933 by 42,151 SNPs, from the environmental isolate BVH88 by 44,773 SNPs, from Z9-6 by 40,994 SNPs, and from the fish isolate BIM B-1809 by 46,976 SNPs. The full phylogenetic tree incorporating all publicly available *A. allosaccharophila* genomes (*n* = 57; Supplementary Figure S1) further confirmed that clinical isolates did not form a monophyletic clade, and that S14 and ATCC 35942 were separated by multiple environmental strains.

## 4 Discussion

This study presents the first comprehensive genomic characterization of *Aeromonas* isolates from diarrheal patients in Beijing using active surveillance, revealing high genetic diversity, emerging chromosomal colistin resistance, species-specific virulence repertoires, and a potential foodborne outbreak cluster.

The isolation rate of 3.3% observed in our study marks a significant increase compared to the previously documented rate of 0.65% in this specific locality during 2016–2022 (Li et al., 2025). This increase should be interpreted with caution, as it reflects both methodological differences and potential temporal changes in prevalence. Methodologically, the earlier study relied on passively collected isolates using standard culture techniques, whereas our study employed active surveillance with APW enrichment followed by PCR screening, which likely enhanced recovery sensitivity. This optimized approach may have substantially contributed to the observed increase, in addition to any genuine temporal changes in prevalence. The species distribution—with *A. veronii* (56.52%) and *A. caviae* (26.09%) predominating—aligns with global epidemiological patterns (Lou et al., 2025; Sadeghi et al., 2024). Of particular interest was the isolation of less frequently encountered species, including *A. enteropelogenes* and *A. allosaccharophila*. The former species demonstrates a rare association with human diarrheal cases. To the best of our knowledge, our study represents the first clinical isolation of *A. allosaccharophila* from human diarrheal specimens in Chinese literature, whose genome has been sequenced and publicly deposited, and one of only a few documented clinical cases of this species worldwide, following the U.S. strain ATCC 35942 (Martinez-Murcia et al., 1992) and the Spanish strain MDC561 (Saavedra et al., 2007). However, the genome of MDC561 is not publicly available, leaving ATCC 35942 as the only comparable clinical genome in public databases.

To understand the global phylogenetic position of the rare *A. allosaccharophila* clinical isolate S14, we performed a comprehensive core-genome SNP comparison with all publicly available genomes of this species. Our core-genome SNP analysis revealed that the Beijing clinical isolate S14 differed from the U.S. clinical strain ATCC 35942 by 42,099 SNPs, from the type strain CECT 4199 by 44,223 SNPs, and from other publicly available *A. allosaccharophila* strains by 40,994 to 46,976 SNPs. Together with ANI values above 95% (Figure 1), these data confirm the species assignment of S14 as *A. allosaccharophila* while demonstrating substantial genetic divergence from all previously characterized strains. This distance is substantially larger than the <100–200 SNP differences typically observed among highly clonal isolates of the same sequence type in *Aeromonas* (Erickson et al., 2023). To provide an additional benchmark, we note that in the well-characterized seventh-pandemic *Vibrio cholerae* phylogeny, a core-genome SNP difference of approximately 20,000 was sufficient to distinguish distinct phylogenetic lineages (Mutreja et al., 2011). The 42,099 SNP distance between S14 and ATCC 35942 is larger, supporting their classification as distinct phylogenetic lineages within the same species. This substantial genetic gap suggests that the two clinical isolates (China and USA) likely diverged from a common ancestor long before their independent associations with human disease, and that *A. allosaccharophila* may harbor multiple distinct clonal lineages capable of causing human gastroenteritis. The absence of a closely related genome in public databases further underscores the rarity and potential geographic restriction of this species. Notably, the full phylogenetic tree including all available genomes (Supplementary Figure S1) showed that clinical isolates did not cluster together, indicating that pathogenicity in humans may not be restricted to a single genetic lineage within *A. allosaccharophila*. This pattern contrasts with some other *Aeromonas* species (e.g., *A. dhakensis*), where certain clones appear to be more frequently associated with human infections (Chen et al., 2016). While our data suggest genetic heterogeneity among human disease-causing *A. allosaccharophila* strains, the scarcity of publicly available clinical genomes (*n* = 2; the U.S. isolate ATCC 35942 and our isolate S14) precludes definitive conclusions. Future studies incorporating additional clinical genomes are warranted to validate this pattern.

A high level of antimicrobial resistance was observed among the *Aeromonas* isolates, with high concordance between phenotypic resistance patterns and their corresponding genetic determinants. The pronounced resistance rate to ampicillin/sulbactam (78.26%) exhibited direct correlation with the widespread presence of *β*-lactamase genes *ampS* and *blaCEPH-A3*. This specific resistance profile (AMP + AMS) maintains consistency with historical local surveillance data (Li et al., 2025), suggesting stable persistence of this resistance pattern within the regional *Aeromonas* population. The clear association between carbapenem resistance phenotypes (ertapenem 21.73%, meropenem 8.70%) and the detection of *carbapenemase* genes (30.43%) indicates these genetic elements serve as the primary mediators of carbapenem resistance.

The chromosomal localization of *mcr* genes in our isolates has important public health implications. Most notably, our study provides the inaugural documentation of *mcr-3.25* and *mcr-3.3* in *Aeromonas* isolates from this geographical region, with high concordance between genotypic detection (8.70%) and phenotypic resistance (8.70%). Using Plasmer analysis, both *mcr* genes were predicted to be located on the chromosome. The chromosomal localization predicted for the two *mcr*-positive isolates in this study provides direct genomic evidence for this chromosomal carriage, although experimental validation (e.g., conjugation assays) would be required to definitively confirm their immobility. Although *mcr* genes were originally described as mobile due to their initial discovery on plasmids, subsequent studies have demonstrated that they can also be located on chromosomes in various bacterial species, including *Aeromonas*. This observation aligns with the emerging understanding that *Aeromonas* species serve as natural reservoirs and evolutionary ancestors of *mcr-3* family genes (Yu et al., 2023; Guo et al., 2024). A study by Yu et al. (2023) demonstrated that nonmobile colistin resistance (*NMCR-3*) determinants are widely distributed on *Aeromonas* chromosomes and represent the ancestral precursors of plasmid-borne *mcr-3* genes that have subsequently disseminated to Enterobacteriaceae (Yu et al., 2023). The chromosomal location of *mcr* genes in our isolates, in the absence of plasmid-associated sequences, suggests that these strains represent part of the natural resistance reservoir rather than cases of recent horizontal acquisition. Applying the *η* risk index (*MCR*/*NMCR*) framework proposed by Yu et al. (2023), which was designed to evaluate the regional propagation potential of *NMCR-3*, our findings from Beijing (where no plasmid-borne *mcr* was detected) align with the observation that Asia currently faces a lower risk of escalating *MCR* incidence compared to North America and Europe (Yu et al., 2023).

The virulence gene distribution in this study reveals clear species-associated patterns alongside notable intraspecies heterogeneity. The virulence gene distribution in this study reveals clear species-associated patterns alongside notable intraspecies heterogeneity. The universal presence of *exeG* (T2SS) and core *flagellar* genes (*fliG*, *fliM*, *flmH*) in all isolates highlights their essential roles in *Aeromonas* secretion and motility, consistent with previous descriptions of these systems (Janda and Abbott, 2010). The *acr*/*exs*-type T3SS was confined exclusively to *A. veronii* (61.5% of isolates) and was absent from all other species. This finding aligns with previous reports that *A. veronii* frequently carries T3SS genes (Silver and Graf, 2009; Chacón et al., 2004). In contrast, the *ats*-type T3SS (marked by *atsA*) was found only in the single *A. dhakensis* isolate, which also uniquely carried the complete RTX toxin cluster and the most extensive adhesin repertoire. This observation is consistent with the recognized high virulence potential of *A. dhakensis* (Chen et al., 2016). The core T6SS gene *hcp* was detected in *A. caviae*, *A. enteropelogenes*, and *A. dhakensis*, but was completely absent from *A. veronii*. This reciprocal distribution—T3SS in *A. veronii* versus T6SS in *A. caviae* and *A. enteropelogenes*—represents a clear genomic pattern. A plausible biological interpretation of this pattern is that *A. veronii* may rely on T3SS-mediated direct effector injection into host cells, whereas *A. caviae* and *A. enteropelogenes* may employ T6SS for bacterial competition and phagocyte evasion, but direct experimental evidence would be needed to confirm these functional predictions in the context of *Aeromonas* pathogenesis. A recent global genomic analysis of *A. caviae* revealed that strains carrying T6SS are more common in human gastroenteritis than in environmental sources and are often phylogenetically related (Chong et al., 2024), further supporting the clinical relevance of T6SS in this species. The limited virulence repertoire of *A. allosaccharophila* is consistent with its rare clinical isolation and mild disease presentation. Despite lacking most major virulence systems, the isolation of this species from a diarrheal patient warrants attention as it may harbor uncharacterized virulence determinants and provides an opportunity to explore the genomic population structure of *A. allosaccharophila*. Importantly, substantial intraspecies heterogeneity exists, particularly within *A. veronii*, where five of 13 isolates carried neither T3SS nor T6SS. This finding emphasizes that species identification alone is insufficient to predict virulence potential; direct genotyping of virulence genes, such as detection of *acr1* or *hcp*, is recommended for risk assessment in clinical settings.

Phylogenetic reconstruction revealed substantial genetic diversity among isolates, which segregated into distinct species-specific clades with 22 unique sequence types identified among 23 isolates. From a genomic perspective, we identified a tightly clustered monophyletic group comprising three *A. veronii* isolates with minimal SNP differences (9–27). From a temporal perspective, These isolates were recovered within a narrow 96-h temporal window from patients without documented epidemiological connections. Together, these observations suggest a possible cluster that would have remained undetected through conventional surveillance methods. However, we acknowledge that confirmatory epidemiological evidence (e.g., food exposure interviews, common source investigation) would be needed to establish a definitive transmission link. This finding demonstrates the utility of genomic surveillance for generating hypotheses about potential transmission events in foodborne pathogen monitoring, which can then be investigated through targeted epidemiological follow-up.

Several limitations warrant acknowledgment. The sample size is moderate and derived from a single geographical area, which may limit generalizability. The suspected outbreak cluster lacked detailed epidemiological confirmation (e.g., food exposure interviews). For the *mcr* genes, we did not perform conjugation experiments to assess transferability. Furthermore, the role of *Aeromonas* in enteric disease remains controversial given the possibility of asymptomatic carriage, and our study did not include healthy controls. While the detection rate of 3.3% in diarrheal patients is consistent with previous reports from diarrheal populations, the absence of controls means we cannot definitively attribute causation. Additionally, while several *A. allosaccharophila* strains have been reported in the literature (e.g., MDC561 and the Chinese leech isolate WP204), their genome sequences are not publicly available, which restricted our global comparison to only a subset of available strains. Future efforts to sequence and release these genomes would greatly enhance our understanding of this species’ population structure and pathogenic potential. Future research should encompass expanded surveillance scope, integrated epidemiological investigations combined with genomic data, and functional studies of *mcr* mobilization potential to comprehensively elucidate *Aeromonas* transmission dynamics and clinical impacts.

## Data Availability

The genome sequences of the *Aeromonas* strains sequenced in this study have been deposited at GenBank/DDBJ/ENA under the BioProject ID no. PRJNA1467509.

## Footnotes

1
https://pubmlst.org/organisms/Aeromonas-spp

2
http://tnpedia.fcav.unesp.br
