# Supplementary material for: Genomic insights into Aeromonas infections in diarrheal patients: high diversity, emerging resistance, and potential outbreak in Beijing
Source: Front Microbiol. 2026 Aug 24;17:1909810. doi: 10.3389/fmicb.2026.1909810 (PMC13547312; doi:10.3389/fmicb.2026.1909810)
Supplement: Supplementary file 1 [file Supplementary_file_1.docx]

Supplementary Material

# Supplementary Materials

Supplementary Table S1. List of all publicly available *Aeromonas* *allosaccharophila* genomes used in this study, including strain name, source, isolation location, assembly level, and NCBI accession number.

| strain name | NCBI accession number | Isolation source | Geographic location | assembly level |
| --- | --- | --- | --- | --- |
| 12-27LHC4-1 | GCA36991045.1 | chicken raw meat | China:Chengdu | Scaffold |
| ABBC-1 | GCA48551555.1 | roreshore beach sand | USA: Wisconsin | Contig |
| CECT4911 | GCA18359965.1 | feces | Switzerland | Contig |
| INSAq178 | GCA21441165.1 | fish | Portugal | Scaffold |
| FUJ01747 | GCA35203845.1 | wound | Japan: Tokyo | Contig |
| Aer19_25 | GCA53376235.1 | raw sewage | Brazil: Sao Paulo, Sao Paulo | Contig |
| 12-27GPP17-2 | GCA36991085.1 | pork raw meat | China:Chengdu | Scaffold |
| GRABPA_AC_28 | GCA54864315.1 | wastewater | Chile: Santiago | Contig |
| FUJ01819 | GCA35205425.1 | bile | Japan: Chiba | Contig |
| Aer67_25 | GCA53373475.1 | raw sewage | Brazil: Sao Paulo, Sao Paulo | Contig |
| GRABPA_AC_37 | GCA54864175.1 | wastewater | Chile: Santiago | Contig |
| 12-27LHP1-2 | GCA36991005.1 | pork raw meat | China:Chengdu | Scaffold |
| D_26 | GCA50431125.1 | aquatic products | China: Chengdu | Contig |
| Aer_89 | GCA51661285.1 | meat | Brazil: Sao Paulo, Sao Paulo | Contig |
| Aer24_25 | GCA53373135.1 | raw sewage | Brazil: Sao Paulo, Sao Paulo | Contig |
| 0057-6 | GCA40670695.1 | food | Canada: Ottawa | Contig |
| D_156 | GCA50431685.1 | aquatic products | China: Chengdu | Contig |
| Z9-6 | GCA3265495.1 | chicken meat | China | Scaffold |
| Z9-6 | GCA900491725.1 | chicken meat | China | Scaffold |
| TTU2014-159ASC | GCA1446895.1 | feces | USA: Texas Panhandle | Contig |
| D_49 | GCA50430625.1 | aquatic products | China: Chengdu | Contig |
| D_36 | GCA50430905.1 | fish | China: Chengdu | Contig |
| Aer30_25 | GCA53376215.1 | raw sewage | Brazil: Sao Paulo, Sao Paulo | Contig |
| FDAARGOS_933 | GCA16026615.1 | not applicable | USA | Complete Genome |
| D3 | GCA42863415.1 | aquatic sediment | Australia | Contig |
| WFFMC_MAG_664 | GCA51651385.1 | fish gut | China: Yalong River | Contig |
| ATCC 35942 | GCA6246445.1 | feces | USA: South Carolina | Scaffold |
| not collected | GCA963883035.1 | not collected | not collected | Scaffold |
| INSAq241 | GCA21441305.1 | mussels | Portugal | Contig |
| 12-27GPP9-2 | GCA36991125.1 | pork raw meat | China:Chengdu | Scaffold |
| SEPT_LG1 | GCA41429675.1 | unknown | Canada: west of winnipeg | Chromosome |
| CN18A046 | GCA46644485.1 | stool | China: Shaanxi | Contig |
| CN18A420 | GCA46644625.1 | stool | China: Henan | Contig |
| 413150 | GCA40670705.1 | food | Canada: Ottawa | Contig |
| D_155 | GCA50431705.1 | aquatic products | China: Chengdu | Contig |
| 0026-5 | GCA40670915.1 | food | Canada: Ottawa | Contig |
| Aer58_25 | GCA53373535.1 | Raw sewage | Brazil: Sao Paulo, Sao Paulo | Contig |
| M01 | GCA34567995.1 | unknown | China:jiangsu | Complete Genome |
| Ho15-B15L1 | GCA41929115.1 | digestive tract | USA: Storrs | Scaffold |
| CN18A189 | GCA46644665.1 | stool | China: Anhui | Contig |
| BVH88 | GCA820245.1 | Environmental | unknown | Scaffold |
| unknown | GCA963866445.1 | unknown | unknown | Scaffold |
| Aer50_25 | GCA53373575.1 | stool | Brazil: Sao Paulo, Sao Paulo | Contig |
| CCM 4363 | GCA1908545.1 | diseased elver | Spain: Valencia | Contig |
| CECT 4199 | GCA819685.1 | Diseased eel | Valencia: Spain | Scaffold |
| 25-AL00116 | GCA54052725.1 | unknown | France | Contig |
| BIM B-1809 | GCA32849865.1 | carp liver | Russia: Belgorod region | Complete Genome |
| 12-27LMLP7-2 | GCA36990845.1 | pork raw meat | China:Chengdu | Scaffold |
| AE23W20901_35 | GCA46085525.1 | freshwater | China:Zhejiang | Contig |
| AE23W20902 | GCA46085545.1 | freshwater | China:Zhejiang | Contig |
| D_159 | GCA50431625.1 | aquatic products | China: Chengdu | Contig |
| D_133 | GCA50432185.1 | aquatic products | China: Chengdu | Contig |
| AE59-TE2 | GCA10906985.2 | mugil brasiliensis scales | Brazil:Rio de Janeiro | Chromosome |
| K520 | GCA29024985.1 | unknown | China: Zhejiang | Complete Genome |
| KLG2 | GCA901212385.1 | river Don | South Yorkshire | Contig |
| Unknown | GCA963901125.1 | unknown | Unknown | Contig |

Supplementary Table S2. Core-genome SNP distance matrix among S14 and 6 *Aeromonas* *allosaccharophila* strains with publicly available genomes.

|  | S14 | *A. allosaccharophila* CECT 4199 | *A. allosaccharophila* BVH88 | *A. allosaccharophila* ATCC 35942 | *A. allosaccharophila* FDAARGOS_933 | *A. allosaccharophila* BIM B‑1809 | *A. allosaccharophila* Z9‑6 |
| --- | --- | --- | --- | --- | --- | --- | --- |
| S14 | 0 | 44223 | 44773 | 42099 | 42151 | 46976 | 40994 |
| *A. allosaccharophila* CECT 4199 | 44223 | 0 | 45454 | 44241 | 44258 | 47152 | 43865 |
| *A. allosaccharophila* BVH88 | 44773 | 45454 | 0 | 44921 | 44887 | 48348 | 43914 |
| *A. allosaccharophila* ATCC 35942 | 42099 | 44241 | 44921 | 0 | 41155 | 46761 | 40998 |
| *A. allosaccharophila* FDAARGOS_933 | 42151 | 44258 | 44887 | 41155 | 0 | 47497 | 41259 |
| *A. allosaccharophila* BIM B‑1809 | 46976 | 47152 | 48348 | 46761 | 47497 | 0 | 46875 |
| *A. allosaccharophila* Z9‑6 | 40994 | 43865 | 43914 | 40998 | 41259 | 46875 | 0 |

Supplementary Figure S1. Maximum-likelihood phylogenetic tree based on core-genome single nucleotide polymorphisms (cgSNPs) of all publicly available *Aeromonas* *allosaccharophila* strains.

Supplementary Figure S2. Supplementary Figure S2. Schematic comparison of the *mcr*-harboring contigs from strains S6 (A. veronii) and S1. Strain S6 contig (14,370 bp) contains a complete ISAeca1 transposase (IS30 family) at the left end (106–1,053 bp), an *mcr* gene ( 8,894–10,516 bp) in the central region, and another ISAeca1 element at the right end (13,385–14,332 bp). Strain S1 contig (1,468 bp) carries an mcr gene (1–1,073 bp) that aligns to the corresponding region of S6. ISAeca1 transposase elements are shown in green; *mcr* genes in red. Arrowheads (◄/►) indicate gene orientation on the forward or reverse strand.
